# Sex effects on clinical features in *LRRK2 G2385R* carriers and non-carriers in Parkinson's disease

**DOI:** 10.1186/s12868-021-00623-6

**Published:** 2021-03-26

**Authors:** Shi-Shuang Cui, Rao Fu, Juan-Juan Du, Yi-Qi Lin, Pei Huang, Chao Gao, Hai-Yan Zhou, Sheng-Di Chen

**Affiliations:** 1 Department of Neurology & Institute of Neurology, Ruijin Hospital Affiliated To Shanghai Jiao Tong University School of Medicine, 197 Rui jin Er Road, Shanghai, 200025 China; 2 Department of Geriatrics, Ruijin Hospital Affiliated To Shanghai Jiao Tong University School of Medicine, Shanghai, 200025 China

**Keywords:** Parkinson’s disease, Sex, *LRRK2 G2385R*, Clinical feature, Transcranial sonographic

## Abstract

**Background:**

Differences of genotypes between male and female have been studied in Parkinson’s disease (PD), but limited research has focused on the comparison between sexes with *LRRK2 G2385* variant.

**Objective:**

The aim of this study was to explore sex effects in the same genetic subtype and role of leucine-rich repeat kinase 2 (*LRRK2*) *G2385R* variants in the same sex in PD.

**Methods:**

613 PD patients were recruited from the Movement Disorders Clinic in Ruijin Hospital. We did not include healthy controls in this study. The data collected includes demographic information, disease history, scores of motor and non-motor symptoms scales, midbrain transcranial sonography and DNA. Binary logistic regression analysis was performed to evaluate the association between clinical features and sex in *LRRK2 G2385R* carriers and non-carriers, as well as the association between the clinical features and *LRRK2 G2385R* variants in male and female sex.

**Results:**

Sex distribution is similar in *LRRK2 G2385R* carriers and non-carriers. In male sex, *LRRK2 G2385R* carriers showed lower risk in cognitive impairment compared with non-carriers (OR = 0.301, p = 0.003, 95%CI 0.135–0.668). In female sex, *LRRK2 G2385R* carriers showed lower risk in autonomic dysfunction compared with non-carrier (OR = 0.401, p = 0.040, 95%CI 0.167–0.960). In *LRRK2 G2385R* non-carriers, female sex showed lower risk of impairment in activity of daily living (OR = 0.610, p = 0.021, 95%CI 0.400–0.928), excessive daytime sleepiness (OR = 0.555, p = 0.007, 95%CI 0.361–0.853), substantia nigra hyperechogenicity (OR = 0.448, p = 0.019, 95%CI 0.228–0.878), autonomic dysfunction frequency (OR = 0.626, p = 0.016, 95%CI 0.428–0.917) and higher risk in mood disorders (OR = 1.691, p = 0.022, 95%CI 1.078–2.654) compared with male. In *LRRK2 G2385R* carriers, female sex showed a lower risk of autonomic dysfunction (OR = 0.294, p = 0.024, 95%CI 0.102–0.849) compared with male.

**Conclusion:**

In contrast to male PD patients, a more benign disease course was observed in female in both *LRRK2* G2385R carriers and non-carriers. However, sex differences were less notable in PD with *LRRK2* G2385R variants.

## Backgrounds

Sex-related differences in symptomatic and epidemiological features have been studied extensively in Parkinson’s diseases (PD). A higher incidence of PD among men has been reported [1] and different clinical characteristics have been reported by sex in PD. A more rapid progression, more severe motor symptoms, greater rigidity and increased mortality have been described among males, while a more frequent tremor-dominant subtype and a higher risk of dyskinesia and fluctuation were reported among females [2–6]. Several previous studies have investigated potential sex differences in non-motor symptoms (NMS), with less consistent findings. A previous study found that male sex in PD showed a stronger positive significant association in almost all NMS with respect to the general population [7]. Some research found that women were associated with more severe mood disorder [8–10]. Other studies found no significant differences in NMS between male and female [11, 12].

Mutation of the leucine-rich repeat kinase 2 (LRRK2) gene is the most frequent genetic cause of PD, and the G2385R variant is the most common variant observed in Asian [13, 14]. This variant was observed in more than 10% of PD patients in Chinese population but is absent in Caucasian or Jewish patients [13–15]. Some research reported more frequent subtype of postural instability/gait difficulty and motor fluctuation in patients with LRRK2 G2385R variant [15, 16], but no major clinical differences except for the non-significant milder non-motor symptoms were found in PD patients with LRRK2 in most previous studies [13, 17, 18].

Different sex effects have been reported in *LRRK2* carriers. A previous meta-analysis indicated that *LRRK2*-associated PD lacks a sex effect [19]. A recently published study found that patients with *LRRK2 G2019S* variants showed less diversity in phenotypes [20]. Nonetheless limited data are available regarding sex effect related to *LRRK2 G2385R* variants as well as effects of *LRRK2 G2385R* variants in terms of sex. The aim of the present cross-sectional study was to evaluate the possible sex and *LRRK2 G2385R* variant differences in clinical features among PD patients, sex effects with *LRRK2 G2385R* mutations and role of *LRRK2 G2385R* variants in women and men with PD.

## Results

Of the 855 PD patients screened for eligibility, 12 were diagnosed secondary Parkinsonism and atypical Parkinsonism, 7 carried with DBS devices and 233 patients refused genetic analyses and transcranial sonographic (TCS). Finally, 613 PD patients were analyzed, including 337 (55.0%) men and 276 (45.0%) women. *LRRK2 G2385R* variant was found in 79 (12.9%) of them. No sex distribution differences were found between *LRRK2 G2385R* carriers and non-carriers (p = 0.729). The demographic and clinical features of PD patients were shown in Table 1.

**Table 1:** demographic and clinical feature of female and male PD within the same genetic group

	LRRK2 G2385R carriers (N = 79)			LRRK2 G2385R non-carriers (N = 534)		
	Female (N = 37)	Male (N = 42)	P	Female (N = 239)	Male (N = 295)	P
Age	61.3 (11.6)	64.7 (7.5)	0.497	61.5 (10.9)	61.5 (10.3)	0.990
Disease duration	5.2 (3.1)	5.4 (4.1)	0.807	5.0 (4.1)	5.1 (4.5)	0.858
AAO	57.4 (11.8)	59.6 (8.1)	0.342	56.1 (11.2)	56.0 (10.5)	0.900
H-Y stage	1.7 (0.5)	1.8 (0.6)	0.565	1.9 (0.7)	1.9 (0.7)	0.874
MDS-UPDRS II	8.8 (5.5)	10.7 (7.1)	0.189	8.7 (7.3)	9.9 (7.8)	0.063
MDS-UPDRS III	24.7 (18.7)	24.6 (15.9)	0.968	22.1 (17.8)	25.0 (19.2)	0.080
NMSS	24.2 (30.0)	30.6 (32.0)	0.359	28.7 (30.7)	31.0 (33.3)	0.414
MOCA	21.7 (6.9)	24.7 (4.2)	0.046	22.5 (5.3)	23.0 (4.9)	0.398
PDSS	96.5 (49.1)	109.7 (44.1)	0.216	100.7 (46.0)	105.8 (43.0)	0.186
HAMD	5.1 (4.6)	4.3 (3.9)	0.384	5.8 (5.3)	5.3 (5.2)	0.288
HARS	5.2 (5.9)	6.5 (5.1)	0.270	7.2 (6.3)	6.2 (6.4)	0.061
SS-16	5.8 (4.0)	6.8 (3.8)	0.270	6.2 (4.2)	5.8 (3.8)	0.265
RBD-HK	15.2 (16.4)	14.1 (16.5)	0.753	13.7 (16.1)	14.0 (18.3)	0.821
SCOPA-AUT	6.9 (8.7)	9.4 (8.5)	0.192	8.7 (9.0)	10.2 (9.1)	0.055
ESS	5.1 (5.5)	5.8 (5.9)	0.574	4.8 (5.3)	6.6 (6.2)	< 0.001
PDQ-39	13.8 (15.5)	16.7 (18.5)	0.462	18.0 (18.8)	15.9 (17.2)	0.192
SN +	37.5%	38.5%	1.000	28.8%	45.3%	0.031

### *LRRK2 G2385R* effects within the same sex

We stratified the enrolled PD patients by sex and used binary logistic regression to analyze the association between *LRRK2 G2385R* carriers and non-carriers in female and male sex respectively. After adjusting age, disease duration and schooling year, we found that males with *LRRK2 G2385R* carriers showed lower risk in cognitive impairment (OR = 0.301, p = 0.003) as compared with males without *LRRK2 G2385R* non-carriers (Table 2) and the association remained significant after Bonferroni correction. After adjusted by age and disease duration, we found that females with *LRRK2 G2385R* carriers showed lower risk in autonomic dysfunction (OR = 0.401, p = 0.040) as compared with females without *LRRK2 G2385R* non-carriers (Table 2). But the association didn’t survive the Bonferroni correction.

**Table 2:** the association between clinical feature and LRRK2 G2385R in male and female

	Male			Female			P-value (all G2385R carriers vs. all non-carriers)	
	OR (95%CI)	p	Genetic power	OR (95%CI)	p	Genetic power	OR (95%CI)	p
EOPD	0.430 (0.174–1.061)	0.067	0.070	0.870 (0.355–2.130)	0.760	0.007	0.595 (0.316–1.120)	0.108
Advanced stage ^a^	0.487 (0.165–1.443)	0.194	0.247	0.360 (0.102–1.273)	0.113	0.177	0.425 (0.189–0.957)	0.039
Impaired ADL ^a^	1.195 (0.557–2.563)	0.647	0.074	1.228 (0.524- 2.877)	0.637	0.078	1.167 (0.665–2.048)	0.591
Motor dysfunction ^a^	1.540 (0.746–3.177)	0.243	0.247	1.647 (0.739- 3.670)	0.166	0.249	1.549 (0.908–2.642)	0.108
Motor complication ^a^	1.779 (0.688- 4.600)	0.234	0.391	1.372 (0.461–4.088)	0.570	0.132	1.557 (0.763–3.180)	0.224
Cognitive impairment ^b^	0.301 (0.135–0.668)	0.003	0.005	0.709 (0.276–1.820)	0.475	0.058	0.448 (0.248–0.809)	0.008
Sleep disorder ^c^	0.857 (0.312–2.357)	0.765	0.077	1.026 (0.385–2.739)	0.959	0.048	1.084 (1.034–1.136)	0.001
Mood disorder ^c^	0.781 (0.305–1.996)	0.605	0.119	0.425 (0.140–1.284)	0.129	0.169	0.587 (0.288–1.198)	0.143
olfaction ^c^	0.605 (0.291–1.255)	0.177	0.141	1.680 (0.702–4.020)	0.244	0.299	0.976 (0.562–1.695)	0.931
RBD ^c^	0.806 (0.386–1.685)	0.567	0.102	1.123 (0.500–2.520)	0.779	0.062	0.926 (0.539–1.592)	0.781
Autonomic dysfunction ^c^	0.879 (0.439–1.762)	0.717	0.070	0.401 (0.167–0.960)	0.040	0.044	0.653 (0.387–1.102)	0.110
EDS ^c^	0.804 (0.379–2.095)	0.571	0.103	1.637 (0.695–3.854)	0.259	1.637	1.070 (0.611–1.875)	0.813
Life quality ^c^	1.050 (0.526–2.095)	0.891	0.048	0.953 (0.437–2.080)	0.903	0.953	1.001 (0.597–1.678)	0.998
SN + ^c^	0.773 (0.236–2.530)	0.670	0.125	1.602 (0.344–7.455)	0.548	1.602	0.988 (0.388–2.519)	0.980

^a^Adjusted for age, disease duration and LEDD

^b^Adjusted for age, disease duration and years of education

^c^Adjusted for age and disease duration

### Sex effects within the same genetic subtype

Stratified by genotypes and adjusting age and disease duration, female sex showed milder ADL impairment (OR = 0.610, P = 0.021), lower risk of EDS (OR = 0.555, p = 0.007), lower risk of SN + (OR = 0.448, P = 0.019), lower risk of autonomic dysfunction (OR = 0.626, p = 0.016) and higher risk of mood disorders (OR = 1.691, p = 0.022) in non-carriers compared with male in *LRRK2 G2385R* non-carriers (Table 3). In *LRRK2 G2385R* carriers, female sex showed a lower risk of autonomic dysfunction (OR = 0.294, p = 0.024). But these associations didn’t survive after Bonferroni correction.

**Table 3:** the association between clinical feature and sex in LRRK2 G2385R carriers and non-carriers

	LRRK2 G2385R carriers		LRRK2 G2385R non-carriers		P-value (all female PD vs. all male PD)	
	OR (95%CI)	p	OR (95%CI)	p	OR (95%CI)	p
EOPD	1.701 (0.508–5.693)	0.388	0.841 (0.563–1.256)	0.397	0.904 (0.618–1.321)	0.602
Advanced stage ^a^	1.589 (0.222–11.353)	0.644	1.361 (0.824–2.248)	0.229	1.35 (0.838–2.184)	0.216
Impaired ADL ^a^	0.599 (0.183–1.957)	0.392	0.610 (0.400–0.928)	0.021	0.617 (0.416–0.913)	0.016
Motor dysfunction ^a^	0.750 (0.261–2.153)	0.592	0.695 (0.473–1.019)	0.063	0.698 (0.488–0.999)	0.049
Motor complication ^a^	0.840 (0.201–3.513)	0.812	1.259 (0.720–2.199)	0.419	1.193 (0.714–1.995)	0.500
Cognitive impairment ^b^	1.583 (0.458–5.477)	0.468	0.749 (0.485–1.156)	0.192	0.844 (0.564–1.264)	0.411
Sleep disorder ^c^	2.006 (0.525–7.655)	0.309	1.358 (0.830–2.222)	0.223	1.417 (0.893–2.247)	0.139
Mood disorder ^c^	0.854 (0.211–3.459)	0.825	1.691 (1.078–2.654)	0.022	1.594 (1.041–2.441)	0.032
olfaction ^c^	1.850 (0.618–5.537)	0.271	0.686 (0.463–1.016)	0.060	0.774 (0.536–1.116)	0.170
RBD ^c^	1.558 (0.575–4.218)	0.383	1.224 (0.823–1.821)	0.317	1.278 (0.885–1.844)	0.190
Autonomic dysfunction ^c^	0.294 (0.102–0.849)	0.024	0.626 (0.428–0.917)	0.016	0.574 (0.402–0.820)	0.002
EDS ^c^	1.095 (0.383–3.125)	0.866	0.555 (0.361–0.853)	0.007	0.619 (0.417–0.919)	0.017
Life quality ^c^	1.024 (0.394–2.644)	0.961	1.054 (0.722–1.537)	0.786	1.003 (0.986–1.019)	0.764
SN + ^c^	0.980 (0.149–6.443)	0.983	0.448 (0.228–0.878)	0.019	0.488 (0.260–0.919)	0.026

^a^Adjusted for age, disease duration and LEDD

^b^Adjusted for age, disease duration and years of education

^c^Adjusted for age and disease duration

## Discussion

### The effect of *LRRK2 G2385R* on clinical profile in female and male PD

Our study indicated that sex distribution was similar between carriers and non-carriers. A previous multi-center study in China reported similar sex distribution [13]. Consistent with a previous study, most clinical variables were similar in *LRRK2 G2385R* carriers and non-carriers regardless of sex [13]. However, there was two exceptions. In male sex, cognitive impairment was observed less frequent in carriers than non-carriers among PD. Similar with our study, a previous research also observed higher score of Mini-Mental State Examination (MMSE) in *LRRK2 G2385R* carriers than that in non-carriers in China [15]. However, other studies did not find significant differences in dementia between carriers and non-carriers [13, 21]. The lack of association between *LRRK2 G2385R* variants and cognition in female may support that the effect of *G2385R* on cognition existed in some but not all studies. In previous studies, the frequency of autonomic symptoms was similar between *LRRK2*-PD and idiopathic PD [13, 15, 22]. Based on the result of the current study, we also found no significant differences in autonomic dysfunction between *LRRK2 G2385R* carriers and non-carriers. However, in female sex, a lower risk of autonomic dysfunction was found in *LRRK2 G2385R* carriers compared with non-carriers. These results may indicate that the effects of *LRRK2 G2385R* variants may different between sexes.

### The effect of sex on clinical profile in *LRRK2 G2385R* carriers and non-carriers

In the present study, female sex in PD with *LRRK2 G2385R* non-carriers had milder severity in motor symptoms and lower risk in EDS, autonomic dysfunction and SN hyperechogenicity but higher risk in mood disorders, which is in agreement with most published data [3, 10, 23–25]. Sex differences in cognition was inconsistent. Some studies found male sex were at risk, but others found female sex [3, 8, 26, 27]. We also found that male sex among *LRRK2 G2385R* non-carriers had a tendency to develop cognitive impairment. Besides, EDS, the risk factor for cognitive impairment, was observed more frequent in male sex. It may help explain the higher susceptibility of cognitive impairment in male sex. Previous studies discovered significant positive correlations between the frequency of SN + and clinical scores [24, 28, 29]. Consequently, the relatively reserved motor function in female sex may be explained by the similar trends of TCS in our study. Except TCS, the similar trends were also observed by other neuroimaging and fluid biomarker, such as declined brain dopamine binding and lower urate concentrations in male patients [30, 31]. Genetics such as estrogen-related gene or brain-derived neurotrophic factor gene, hormonal influences such as estrogens, immunological factors, environmental exposures, or a combination of these are likely contributors to sex differences in PD via their influence on mitochondrial function, oxidative stress and inflammation [32–36].

However, except for the autonomic dysfunction, we didn’t see any sex effects in PD with *LRRK2 G2385R* carriers, and the mechanism of which is still unclear. One potential explanation is that *LRRK2 G2385R* carriers may have a less heterogeneous phenotypic presentation than non-carriers, and this might mitigate potential sex differences due to *LRRK2 G2385R* variants thus leading to a general tendency to neurodegeneration, which is not influenced by sex [19, 20].

### Strength and limitation

To our best knowledge, this is the first study to study the sex effects on clinical features with and without *LRRK2 G2385R* mutation and the role of *LRRK2 G2385R* on clinical features in terms of sex. Assessments in this study were comprehensive, including motor symptoms, various NMS scales and neuroimaging. Besides, we found that sex behaves differently in different genetic subtype. Further studies in *LRRK2* and sex should consider stratification in design or analysis.

Limitations should be considered in interpreting our findings. We enrolled 613 individuals and only 79 was detected with LRRK2 G2385R mutation. The number of *LRRK2 G2385R* carriers was relatively small. In addition, we only detect *LRRK2 G2385R*, and thus non-carrier might include individual with other genetic mutation. Effects of other SNPs and genes cannot be excluded in our study. Besides, for the convenience of analysis and limitation of data, some continuous variables are being treated as binary outcomes with arbitrary cut-offs, leading to weakened powerfulness. These results became insignificant after Bonferroni correction except lower risk of LRRK2 carriers in cognitive impairment in male patients. The relatively small sample size may account for the insignificance after correction. Thus, a larger sample size is needed in future study. Meanwhile, the genetic power of some results was less than 0.05, indicating, to some extent, a limited accuracy due to the relatively small sample size. Consequently, further study with large sample size and healthy subjects as controls are needed.

## Conclusion

In conclusion, our findings suggested that sex distribution was similar in *LRRK2 G2385R* carriers and non-carriers. A more benign disease course was observed in female sex compared to male sex in both *LRRK2 G2385R* non-carriers and carriers. However, sex differences were less notable in PD with *LRRK2* variants.

## Methods

### Participants

855 PD participants in our study were enrolled between Dec 1, 2015, and Jun 30, 2018 from Movement Disorders Clinic at the Department of Neurology, Ruijin Hospital affiliated to Shanghai Jiao Tong University School of Medicine. All patients were diagnosed with PD by movement disorders specialists, according to the criteria of Movement Disorder of Society [37]. Exclusion criteria included deep brain stimulation, secondary Parkinsonism and atypical Parkinsonism, other movement disorders other than PD, severe hearing or visual loss, inability to speak or write, or other conditions that might interfere with the reliable completion of clinical assessments and patients refused to genetic analysis and TCS. The study was approved by the medical ethics committee of Rui Jin Hospital affiliated to Shanghai Jiao Tong University School of Medicine. Participants gave written informed consent before inclusion in the study.

### Assessments

Demographic including age, sex and years of education were recorded during a clinical interview. Disease-related variables including age at onset (AAO) and disease duration were collected. Disease stage was assessed with the Hoehn &Yahr staging (H-Y stage). Advanced stage was defined as more than or equal to 2.5. Disease-related decline in activity of daily living (ADL) and motor function were assessed with the Movement Disorder Society Unified Parkinson’s Disease Rating Scale (MDS-UPDRS) part 2 and 3 and dichotomized on the sample median. Motor complications of therapy were assessed with MDS-UPDRS part 4. Life quality was assessed by the 39-item Parkinson disease questionnaire (PDQ-39) and dichotomized on the sample median. The equivalent daily dose of L-dopa (mg/day) (LEDD) of dopamine agonists, Catechol-O-methyltransferase (COMT) and Monoamine oxidase (MAO-B) inhibitors was calculated for each patient as previously proposed [38].

Cognitive function was assessed with the Montreal Cognitive Assessment (MoCA) Beijing Version [39]. cut-off value for cognitive impairment was 25/26 and 1-point correction for persons educated no more than 12 years. Depression and anxiety were quantified with the 17-item Hamilton Depression Rating Scale (HAMD) and Hamilton Anxiety Rating Scale (HARS), respectively. Patients with mood disorders was defined with a cut-off of 13/14 in HAMD or 12/13 HARS [40]. Olfactory function was assessed with 16-item odor identification test from the extended version of sniffin' sticks (SS-16) and hyposmia was considered when SS-16 < 8.3 [41]. Autonomic function was assessed with the scale for outcomes in PD for autonomic symptoms (SCOPA-AUT) and dichotomized on the sample median. Sleep quality was assessed by the Parkinson disease sleep scale (PDSS). Poor sleep quality was defined with a cut of 82/83 in PDSS [42]. Rapid eye movement behavior disorder (RBD), and excessive daytime sleepiness (EDS) were assessed with the RBD Questionnaire-Hong Kong (RBD-HK) and Epworth Sleepiness Scale (ESS). RBD was defined that the score of RBD-HK was more than 17 [43] and EDS was defined that the score of ESS was more than 9 [44].

The TCS examinations were performed within one month after the clinical examination. An experienced sonographer who was blinded to the clinical findings of the subjects performed the examination. Through the acoustic bone window, the sonographer detected the echogenicity of the substantia nigra (SN) using a 2.5 MHz sonographic device (MyLab90, ESAOTE, Italy) with a depth of 16 cm and a dynamic range of 45 dB. The SN was scanned through both temporal bone windows in the axial plane. Some subjects showed no identifiable or vague midbrain structures that were insufficient to be quantitatively assessed, and these were excluded from further assessment. After identifying the butterfly shaped hypoechogenic midbrain, which was surrounded by the hyperechogenic basal cistern, the clearest image of the hyperechogenic signals in the SN region was stored. Both sides of SN echogenic areas from stored images were then manually encircled and measured. SN + was defined as the larger SN echogenic areas (SNL) ≥ 18mm^2^ [24].

### Genetic analysis

Peripheral blood samples were collected from all 613 participants, and DNA was extracted from leukocytes using the sodium dodecyl sulfate–proteinase K phenol–chloro-form method. The Primer Premier 5 (version 5.00, PREMIER Biosoft International) was used to design primers for LRRK2 G2385R. Polymerase chain reaction (PCR) was performed under the following cycle conditions: denaturation at 94℃ for 1 min; 35 cycles of denaturation at 94℃ for 30 s, annealing at 60℃ for 30 s, and elongation at 72℃ for 30 s each; and a final elongation step of 1 min at 72℃. After restriction enzyme digestion, the digestion products were separated by polyacrylamide gel electrophoresis (T = 6%, C = 5%). The gel was dyed with 109 Genefinder (Bio-V, Xiamen, China), and the PCR products were visualized under ultraviolet light. Direct DNA sequencing of the PCR product fragments was performed using the 3070xl automated DNA analyzer (Applied Biosystems, Foster City, CA, USA).

### Statistical analysis

Statistical analyses were performed with SPSS Statistics (version 20.0, SPSS Inc., Chicago, IL, USA). Continuous variables were given as means and standard deviation. Categorical variables were summarized by percentages. Chi square test was performed to test distribution differences between sex and *LRRK2 G2385R* variant. In order to explore the degree to which sex differences were related to gene effects, two stratified analyses were performed. To determine the sex differences within genetic groups, binary logistic analysis was performed to evaluate the possible association between sex stratified by *LRRK2 G2385R* variants. To assess *G2385R* effects within male or female sex, binary logistic analysis was performed between *G2385R* carriers and non-carriers stratified by sex. Disease stage, ADL, motor dysfunction and motor complication were adjusted for age, disease duration and LEDD. The remaining variables were adjusted for age and disease duration except that cognition adjusted for age, disease duration and schooling year. Odds Ratio (OR), 95% Confidence Interval (CI), and p-value (two-tailed test) were computed. Significance of differences was defined as two-tailed p < 0.05. Due to multiple comparisons of logistic regression, the P value was corrected with Bonferroni correction to reduce the false positive. The genetic power was calculated by Power and Sample Size Calculations (Version 3.1.2) [45, 46].

## Data Availability

All data generated or analysed during this study are included in this published article.

## Abbreviations

AAO: Age at onset
ADL: Activity of daily living
CI: Confidence interval
EDS: Excessive daytime sleepiness
ESS: Epworth Sleepiness Scale
HAMD: 17-Item Hamilton Depression Rating Scale
HARS: Hamilton Anxiety Rating Scale
H-Y stage: Hoehn &Yahr staging
LRRK2: Leucine-rich repeat kinase 2
MDS-UPDRS: The Movement Disorder Society Unified Parkinson’s Disease Rating Scale
MMSE: Mini-mental State Examination
MoCA: Montreal Cognitive Assessment
NMS: Non-motor symptoms
OR: Odds ratio
PCR: Polymerase chain reaction
PD: Parkinson’s diseases
PDQ-39: 39-Item Parkinson disease questionnaire
PDSS: Parkinson’s disease sleep scale
RBD: Rapid eye movement sleep behavior disorder
RBD-HK: Rapid Eye Movement sleep behavior disorder questionnaire-Hong Kong
SCOPA-AUT: Scale for outcomes in Parkinson's disease for autonomic symptoms
SD: Standard deviation
SN: Substantia nigra
SS-16: 16-Item odor identification test
TCS: Transcranial sonographic
